# Bilateral same-session flexible ureteroscopy for renal stones: a feasible method

**DOI:** 10.25122/jml-2021-0385

**Published:** 2022-02

**Authors:** Bogdan Geavlete, Razvan-Ionut Popescu, Razvan Multescu, Valentin Iordache, Gelu-Adrian Popa, Dragos Georgescu, Petrisor Geavlete

**Affiliations:** 1. Department of Urology, Sanador Hospital, Bucharest, Romania; 2. Department of Urology, Sf. Ioan Clinical Emergency Hospital, Bucharest, Romania; 3. Department of Radiology and Medical Imaging, Sf. Ioan Clinical Emergency Hospital, Bucharest, Romania

**Keywords:** bilateral ureteroscopy, flexible ureteroscopy, renal stone, Holmium laser

## Abstract

A staged ureteroscopic procedure is generally preferred to treat bilateral renal stones. In this study, we evaluated the feasibility of bilateral same-session flexible ureteroscopy (BS-fURS) in renal stones. A total of 81 patients underwent bilateral BS-fURS between March 2014 and March 2021 for bilateral renal stones. The mean stone burden per patient was 17±4 (range 7–27 mm). The average stone density was 1240 HU (970 to 1510). We used 4 types of ureteroscopes: Olympus URF-V2 (34 cases), Storz Flex X2 (30 cases), single-use PUSEN PU 3022 (12 cases), and single-use PUSEN – PU 3033A (5 cases). We specifically set our Holmium laser for dusting, pop-corning, and fragmenting. We found 31 calcium oxalate monohydrate cases, 11 calcium oxalate dehydrate cases, 17 uric acid cases, and 22 magnesium ammonium phosphate cases. The mean operating time was 77 min. (range 52 to 85) for both renal units. The SFRs were evaluated between 1 and 3 months with computed tomography (fragments >3 mm were defined as residual). Double J stenting (6Fr.) was applied bilaterally in 8 cases (9.87%) and unilateral in 34 cases (41.97%). The overall SFRs after 1 and 2 procedures were 81.48% (66/81 cases) and 92.59% (75/81 cases), respectively. Postoperative complications after an overall 96 procedures were Clavien I-II (18.75%) and Clavien III (3.12%). Urinary tract infections were observed in 13 cases (16.04%) without any case of urosepsis. Our experience suggested some BS-fURS advantages as a single anesthetic session and potentially reduced cost associated with treatment. BS-fURS seem feasible, especially for medium-sized bilateral renal stones in high-volume centers.

## Introduction

Urolithiasis represents an important healthcare problem with a major impact on socio-economical aspects and a rapidly increasing incidence and prevalence. The estimated incidence for renal lithiasis reaches a peak value between 30 to 40 years old, and recent studies revealed an estimated risk of stone formation around 11% in males and 7% in females [1, 2]. Moreover, the incidence of the newly diagnosed bilateral stone disease has an increasing trend and 15% of patients have both renal units simultaneously affected [3]. Surgical treatment for renal stone disease is one of the most frequent therapeutic procedures performed by urologists worldwide. The main minimal invasive treatment options for stone disease management include shockwave lithotripsy (SWL), ureteroscopy (URS), percutaneous nephrolithotomy (PCNL), or sometimes combinations between them [4, 5]. Some recent studies reveal a decline for SWL as a preferred treatment practice and an increasing preference for URS [5].

Recent advancements and achievements in developing new endoscopic equipment, especially in flexible ureteroscopy, made this technology a feasible choice for patients with special characteristics like coagulopathy, renal malformations, excessive obesity, SWL failure, or solitary kidney [6–8]. Studies describe an increasing trend for simultaneous bilateral endoscopic surgery (SBES), and flexible ureteroscopy (fURS) appears to become a more suitable treatment method for this kind of pathology compared to PCNL [9]. According to reported complication rates, fURS registered an overall 7.4% in a large multi-center study, recommending this technique as a large high utility procedure for urologists [10].

Besides therapeutic aspects, simultaneous bilateral endoscopic surgery presents other important benefits, including reduced hospital stay, treatment costs, and avoiding second anesthesia for the patient [11]. Simultaneous approaches in bilateral renal stone disease for selected cases associate a decreased patient morbidity, and some small series of studies support this idea and reveal the benefits of safety-effectiveness correlation [12, 13].

This study evaluates the feasibility of simultaneous flexible ureteroscopy for medium to large bilateral stone disease by using recent technological achievements in this field and summarizing their benefits.

## Material and methods

We retrospectively analyzed 81-patient series diagnosed with bilateral stone disease, treated using flexible ureteroscopy in the urology department of Sanador Hospital and Emergency Clinical Hospital Sf. Ioan, Bucharest, Romania, during March 2014 and March 2021.

**Figure 1.**
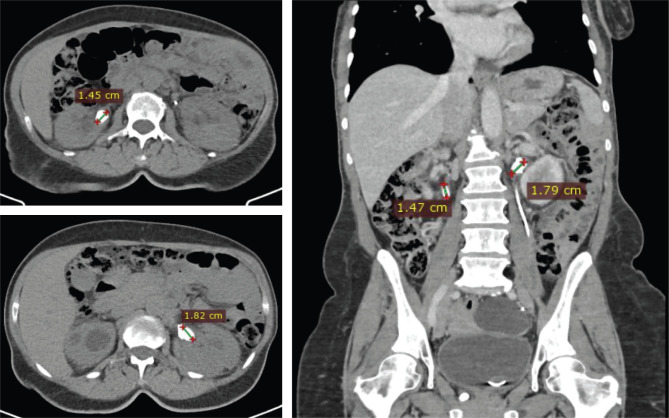
CT sections measuring different dimensions of bilateral renal stones of the same case.

**Figure 2.**
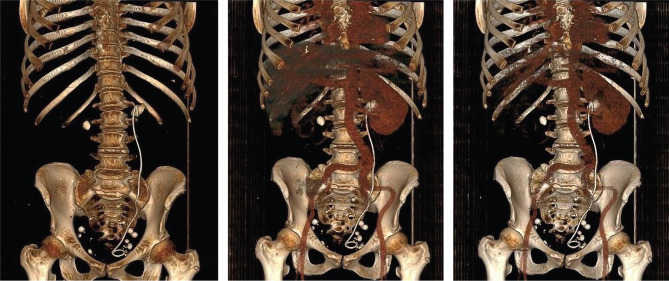
CT 3D reconstruction of bilateral stone disease located in the renal pelvis and left double J stent placement.

Medical records and medical imaging descriptions from the radiology department archive were carefully analyzed to obtain the accurate stone location, dimensions, and density (Figures 1 and 2). The stone burden was estimated using the largest diameter, and the surface area was determined using the standardized formula: SA=l ^×^ w ^×^ π ^×^ 0.25. In the case of one side multiple stone, the stone burden was calculated by summing the largest diameter of each piece. The radiodensity characteristics were estimated after measuring the number of Hounsfield Units (HU) and then separately registered for each case. The stone-free rate was determined using computed tomography between 1 to 3 months after the intervention, and residual fragments were defined as stones bigger than 3 mm.

Inclusion criteria were patients over 18 years old and diagnosed with bilateral renal lithiasis. Exclusion criteria were: pediatric patients, untreated coagulopathies, renal abnormalities (ectopic pelvic kidney, horseshoe kidney), ureteral stone location, stone located in a calyceal diverticulum.

The chemical structure of the stone was determined as part of the complete lithiasis treatment protocol for each patient using infrared spectroscopy.

### Surgical equipment and specifications

All the procedures were performed following a standard protocol which included lithotomy position and spinal anesthesia according to the surgical team experience.

The surgical equipment included semi rigid ureteroscopes, access sheath, flexible ureteroscopes, laser fibers, baskets, and double J stents. The access sheath was placed after performing semirigid ureteroscopy. In cases where access sheath insertion was not possible, a small diameter flexible ureteroscope was preferred to directly access the collecting system. Double J stent insertion was used, when necessary, after the first or second procedure, using direct fluoroscopy guidance. In cases of multiple stone fragments placed on one side, we selected only where the maximum estimated stone burden was below 20 mm.

Fragmentation technique and selection preference for the first renal unit to start the procedure was made after assessing symptomatology, obstruction, or dimension. When neither symptomatology nor obstruction was present, the biggest stone was preferred to start the procedure.

Tables 1 and 2 reveal the types of flexible ureteroscopes and laser settings used in different situations to perform and complete interventions.

**Table 1.** Ureteroscopes. Types and characteristics.

**Type**	**Diameter (Fr.)**	**Nr. of cases**
**Olympus URF-V2**	8.5	34
**Storz Flex X2**	8.4	30
**PUSEN PU 3022**	9.5	12
**PUSEN PU 3033A**	7.5	5

**Table 2.** Holmium laser settings.

	**Energy (J)**	**Frequency (Hz)**	**Pulse**
**Dusting**	Low: 0.5	Hight: 50	Long pulse
**Pop-dusting**	Medium: 0.5–1	Hight: 50	Long pulse
**Pop-corning**	High: >1	Medium: 10–50	Long pulse
**Fragmenting**	High: >1	Low: <10	Short pulse

### Data analysis

CT scans were evaluated and processed using RadiAntViewer to calculate stone dimensions, stone area, and estimated Hounsfield Units. Data obtained from the medical records were registered and processed using Microsoft Excel and Word available on Microsoft Office 18.2008.12711.0 version.

## Results

Demographic data analysis revealed a mean age of 47.7 years old and a male: female ratio of 2.16:1. The mean calculated body mass index (BMI) 27.2 revealed a predisposal for stone formation associated with being overweight. Obesity with BMI exceeding 30 was found in 17 cases.

Urinary tract infections were observed in 13 cases (16.04%) and were previously treated according to antibiogram results. Some of these infections may be related to previous surgical procedures such as double J stenting or cystoscopy performed in other centers or the same institution as an emergency. There were 7 cases in which previously negative urine culture could not be obtained, so it was performed under antibiotic protection administered one day before intervention. No case of urosepsis was registered during the investing period.

The stone chemical structure revealed an increased number of calcium oxalate monohydrates. Secondly identified stones were magnesium ammonium phosphate followed by uric acid and calcium oxalate dihydrate. The mean stone burden was 17±4 mm (range 7–27 mm), and the mean estimated stone surface area (SA) was 181±42 mm^2^ (range 78–291 mm^2^). Stone density varied between 970 and 1510 Hounsfield Units, as shown in Table 3.

**Table 3.** Stone characteristics.

		Nr.	Mean	Range
**Chemical structure**	Calcium oxalate monohydrate	31		
	Calcium oxalate dehydrate	11		
	Uric acid	17		
	Magnesium ammonium phosphate	22		
**Stone burden**			17±4 (mm)	7–27 (mm)
**Radiodensity (Hounsfield Units)**			1240 (HU)	970–1510 (HU)

Operative time and hospital stay were noted in Table 4. The mean operative time for each session was 77 min. Holmium laser was used following the manufacturer’s recommended settings, which was completely exposed in the methodology section.

**Table 4.** Operative Characteristics.

	**Mean**	**Range**
**Operating time**	77 (min.)	52–85 (min.)
**Fluoroscopy time**	58 (sec.)	44–76 (sec.)
**Hospitalization**	44±5 (hours)	26–62 (hours)

Stenting using double J catheters (6 Fr.) was applied, when necessary, as follows: bilateral in 8 cases (9.87%) and unilateral in 34 cases (41.97%). Double J indwelling after the second session of flexible ureteroscopy was not necessary.

**Figure 3.**
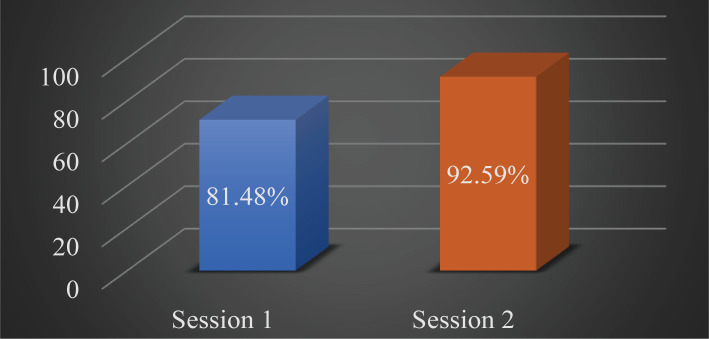
Stone free rate.

**Figure 4.**
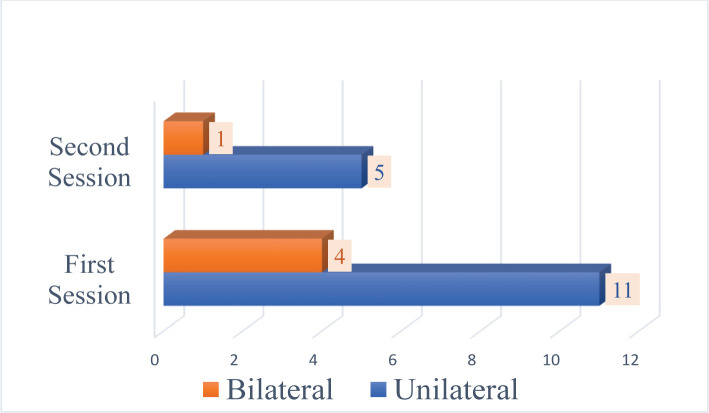
Residual fragments (nr. of cases).

The overall stone-free rate was 81.48% (66 cases) after the first session of simultaneous bilateral flexible ureteroscopy and 92.59% (75 cases) after the second look (Figure 3). After the first session, there were 15 cases with residual stone fragments. Eleven (13.58%) cases were unilaterally residual fragments, and 4 (4.93%) were bilaterally placed. The maximum stone diameter registered as residual was 5 mm. After a second look, 5 (6.17%) cases of unilaterally residual stones remained and 1 (1.23%) bilateral (Figure 4). The residual stones were mostly observed on one side or were related to incomplete elimination after fragmentation.

**Figure 5.**
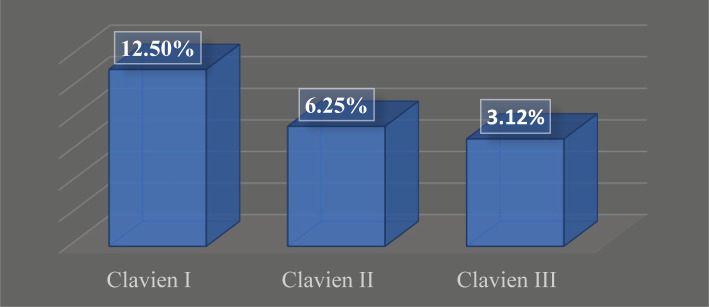
Complications. Clavien-Dindo scale.

Intraoperative complications were represented mainly by mild ureteral injuries (16 cases) and small perforation of the pyelocaliceal system (1 case). Complications were evaluated using the Clavien-Dindo modified system for urological procedures. Postoperative complications graded as Clavien I and II represented 18.75% and Clavien III 3.12% after performing 96 interventions during Session 1 and 2 (Figure 5). There were no cases registered with Clavien IV or V. There were minimal complications registered, including Clavien I and II, mostly related to postoperative pain or fever, and appropriate modifications of the treatment scheme successfully resolved these issues. There were 12 (12.5%) cases of postoperative pain included in Clavien I and 6 (6.25%) cases of postoperative fever and mild hematuria, graded Clavien II. Clavien III complications referred to double J stenting or repositioning under local anesthesia. All patients resumed a normal course after proper medical care.

## Discussion

Flexible ureteroscopy demonstrated its utility and gained increased attention as a preferred therapeutic method for endourological management of single or multiple intrarenal stones [7, 14–16]. Even though the benefits of retrograde intrarenal stones were exposed in many scientific publications, there are still few reports on using this technique as a treatment option for simultaneous surgery on both renal units [17–19]. The present paper reveals the safety and effectiveness of 4 different types of flexible ureteroscopes used for simultaneous endourological treatment in renal stone disease.

The American Urological Association guidelines recommend SWL as an alternative for first-line treatment in renal stones smaller than 20 mm [20]. SWL represents one of the most non-invasive procedures in approaching renal calculi, and its benefits were well stated over time. Short convalescence, lack of anesthesia, and no hospitalization requirement are some of the major advantages of this procedure. The reported stone-free rate varies from 80% to 88% [21], but these results are highly dependent on several factors such as stone location, size, density. The stone-free rate decreases to less than 70% when this approach is used for stones located in the lower pole [22]. Furthermore, the success in obtaining stone-free status decreased to less than 50% when targeting multiple intrarenal calculi [23]. Some studies revealed 63 to 85% of renal injuries diagnosed after CT scan or IRM in patients previously treated using SWL [24].

The reported stone-free rate for PNL reveals excellent values varying from 88% to 100% for renal calculi [21]. When investigating the safety-effectiveness relation, the invasiveness of PNL reveals a low percentage of complications but with a presumed major impact on patients’ condition [20]. Generally, PNL complications are related to the percutaneous access tract and include hemorrhage, perforations of the collecting system, and hydrothorax [25]. Although PNL associates a 95% stone-free rate from the first procedure [26], accessing bilateral renal units may severely affect parenchyma and increase complication rates directly related to this maneuver [27].

The technological advancement in improving flexible ureteroscopes and intracorporeal lithotripsy using holmium laser already acknowledged this technique as an equivalent or superior option to SWL for small calculi [28]. Currently, retrograde intrarenal surgery allows accessing intrarenal stone by using an access sheath that facilitates ureteroscope passage and fragments removal under low pressure of water flow [29]. Nitinol baskets improve stone relocation from difficult positions of the renal collecting system, and holmium laser fibers increase fragmentation efficiency [14].

In the present study, the overall stone-free rate was 81.5% after the first session and 92.6% after the second approach for stone burden more than 21 mm. These data are comparable to those revealed by Portis AJ *et al.* in their study [30]. A recent study on bilateral approach conducted by Huang Z *et al.* revealed an overall 92% stone-free rate after sessions 2 and 3. For the stone burden of more than 20 mm, the same study revealed an 85.7% stone-free rate [31].

Flexible ureteroscopy is an endoscopic procedure that follows the ureteral and intrarenal anatomy, and decreasing the size of the instruments permitted surgeons to obtain low complication rates [32]. Revealing these low levels of complications, flexible ureteroscopy extended intrarenal surgery for special conditions such as coagulopathy, obesity, and pregnancy and overpassed other surgical techniques such as PNL or SWL [33]. In their study regarding simultaneous bilateral ureteroscopy on 71 patients, Watson *et al.* reported an overall postoperative complication rate of 9.7% [17]. Another study conducted by El-Hefnawy *et al.* compared 89 cases of same-session bilateral intrarenal approach and 105 unilateral and revealed no differences between the studied groups regarding complication rate (9.6% *vs.* 6.7%) (18). A smaller study that compared 3 different groups of patients (18 cases of bilateral same session ureteroscopy, 15 cases of stage bilateral ureteroscopy, and 18 cases of unilateral ureteroscopy) revealed equivalent percentages of complication rate between the studied groups [19]. In the present study, minor Clavien I and II complications occurred in 18.75% and Clavien III in 3.12%. There were no Clavien IV and V complications registered. Clavien III complications were generally related to double J stenting or repositioning after the procedure. Compared to other studies, the overall percentage seems to increase, but there were mostly minor complications related to drug administration changes such as vomiting, temporarily increased body temperature, or pain. All Clavien III complications were graded as IIIa because they required only local anesthesia. One of the first prospective studies conducted by Danilovic *et al.* compared the outcomes of bilateral same-session retrograde intrarenal surgery with a unilateral approach and reported a 30.4% Clavien I complication rate. Compared to the present study, Clavien III complications reported by Danilovic were almost equal (4.4%) [34]. Even though there were 29 documented cases of urinary tract infections, the proper antibiotic treatment prevented any cases of postoperative urosepsis.

In many cases, a complete dusting of the renal stones during interventions allowed not to use ureteral double J stents after the procedure. This increased patient quality of life and favored rapid hospital discharge. However, this study presents some limitations. The main limitations refer to the small group of patients and the retrospective analysis of this series of cases.

## Conclusions

Flexible ureteroscopy with holmium laser could be considered a feasible treatment approach for same session bilateral intrarenal stones. BS-fURS seems feasible, especially for medium-sized bilateral renal stones in high-volume centers. Complete dusting of the stones could avoid patient discomfort with ureteral stents. Proper selection of patients, extending surgery on the second side only when the first side was uneventful, represented the keys to success.

## Acknowledgments

### Conflict of interest

The authors declare no conflict of interest.

### Ethical approval

This study was approved by the Ethics Committee of the Sanador Hospital and Sf. Ioan Clinical Emergency Hospital, Bucharest, and was performed under the ethical standards as laid down in the 1964 Declaration of Helsinki and its later amendments (approval ID: 12/07.02.2014).

### Consent to participate

Informed written consent was provided by each participant.

### Availability of data and materials

The datasets generated during and/or analyzed during the current study are available from the authors on reasonable request.

### Authorship 

GB contributed to conceptualizing, methodology, data curation, and editing the manuscript. PRI contributed to writing the original draft, editing, data collection, data curation, and data analysis. MR contributed to data collection, data curation, and data analysis. IV contributed to data collection and data analysis. PGA contributed to data collection and editing the manuscript. GD contributed to data analysis, methodology, and editing of the manuscript. GP contributed to conceptualizing, methodology, and to editing the manuscript.
